# High-fat diet accelerates progression of osteoarthritis after meniscal/ligamentous injury

**DOI:** 10.1186/ar3529

**Published:** 2011-12-07

**Authors:** Robert A Mooney, Erik R Sampson, Jaclyn Lerea, Randy N Rosier, Michael J Zuscik

**Affiliations:** 1 Department of Pathology and Laboratory Medicine, Box 626, University of Rochester Medical Center, 601 Elmwood Avenue, Rochester, NY 14642, USA; 2 Center for Musculoskeletal Research, Box 665, University of Rochester Medical Center, 601 Elmwood Avenue, Rochester, NY 14642, USA

## Abstract

**Introduction:**

Increasing obesity and type 2 diabetes, in part due to the high-fat (HF) Western diet, parallels an increased incidence of osteoarthritis (OA). This study was undertaken to establish a causal relation between the HF diet and accelerated OA progression in a mouse model and to determine the relative roles of weight gain and metabolic dysregulation in this progression.

**Methods:**

Five-week-old C57BL/6 mice were placed on HF (60% kcal) or low-fat (lean, 10% kcal) diets for 8 or 12 weeks before transecting the medial collateral ligament and excising a segment of the medial meniscus of the knee to initiate OA. One group was switched from lean to HF diet at the time of surgery.

**Results:**

Body weight of mice on the HF diet peaked at 45.9 ± 2.1 g compared with 29.9 ± 1.8 g for lean diets, with only those on the HF becoming diabetic. Severity of OA was greater in HF mice, evidenced by the Osteoarthritis Research Society International (OARSI) histopathology initiative scoring method for mice and articular cartilage thickness and area. To assess the importance of weight gain, short- and long-term HF diets were compared with the lean diet. Short- and long-term HF groups outweighed lean controls by 6.2 g and 20.5 g, respectively. Both HF groups became diabetic, and OA progression, evidenced by increased OARSI score, decreased cartilage thickness, and increased osteophyte diameter, was comparably accelerated relative to those of lean controls.

**Conclusions:**

These results demonstrate that the HF diet accelerates progression of OA in a type 2 diabetic mouse model without correlation to weight gain, suggesting that metabolic dysregulation is a comorbid factor in OA-related cartilage degeneration.

## Introduction

Decreased physical activity and the consumption of a high-fat Western diet have contributed to a worldwide epidemic of obesity and associated type 2 diabetes [1]. As a result, the incidence of cardiovascular disease, hypertension, cancer, nonalcoholic fatty liver disease, stroke, and other secondary complications is anticipated to be negatively affected [2,3]. One additional consequence of this epidemic is an increased incidence of osteoarthritis (OA) in both weight-bearing and non-weight-bearing joints [4,5], with more than 50% of those with diabetes having some form of arthritis [4]. In the United States, 52.1% of patients receiving knee replacements in 2005 were obese [6]. In Canada, 87% were either obese or overweight [7]. A morbidly obese patient is 33 times more likely to require knee replacement than is an individual of normal body mass [7]. Despite these statistics, current knowledge does not adequately explain the relative contribution to OA of biomechanical changes resulting from increased body mass versus the metabolic dysfunction in obesity. Although it is widely accepted that the increased biomechanical stresses on the weight-bearing diarthrodial joints due to obesity can initiate and accelerate the OA disease process [8], the potential influence of metabolic dysfunction on the progression of OA is receiving increasing attention [9,10].

Increased dietary fat, as consumed in Western diets, can contribute to both obesity and the metabolic dysfunction that is associated with insulin resistance/type 2 diabetes. Surplus dietary fat is deposited in adipose tissue as well as in skeletal muscle, heart, and liver. As this fat burden increases, adverse effects on systemic metabolism increase [11]. The current model of insulin resistance of obesity as a proinflammatory state is based in part on the observation that adipose tissue in obese individuals and animal models contains increased numbers of activated macrophages that release proinflammatory cytokines such as interleukin-1, interleukin-6, and tumor necrosis factor-α [12,13]. These cytokines act both locally on adipocytes and systemically to impair insulin action in insulin-target tissues [11,14,15]. Metabolism of the abundant circulating free fatty acids in the high-fat diet can also lead to oxidative stress in cells, formation of lipid peroxides, and subsequent oxidative damage to DNA, RNA, and proteins [16-18]. Oxidative stress has been more specifically shown to inhibit insulin-receptor signaling pathways in cells, at least in part through activation of the stress kinases JNK and p38 and to impair mitochondrial function [19,20]. The net effect of the proinflammatory state and tissue oxidative stress is a systemic metabolic dysfunction that defines the metabolic syndrome and type 2 diabetes that are associated with high-fat diets and obesity.

Although it is widely accepted that the increased biomechanical stress associated with obesity contributes to the initiation/progression of OA [21-23], the importance of type 2 diabetes-related systemic metabolic dysregulation in the OA disease process has not been delineated. To begin to address this question, an animal model was used to test the hypothesis that HF diet-induced obesity and associated glucose intolerance/insulin resistance accelerate the progression of osteoarthritis after meniscal/ligamentous injury (MLI). This model was subsequently used to determine whether marked obesity is required for the HF diet to accelerate OA.

## Materials and methods

### Animals

Male C57BL/6J mice purchased from Jackson Laboratories (Bar Harbor, ME) were housed five per cage in a microisolator room on a 12-hour light/dark cycle at the University of Rochester. Mice were placed on a high-fat (60% kcal, D12492) or low-fat (10% kcal, D12450B) diet at 5 weeks of age (Open Source Diets, Research Diets Inc., New Brunswick, NJ). After 8 or 12 weeks on the diet, mice were anesthetized by using intraperitoneal (IP) injection of 60 mg/kg ketamine and 4 mg/kg xylazine, and the medial collateral ligament of the right hindlimb was transected, and a segment of the medial meniscus detached and excised by using a surgical method [24] that we have established in our laboratory [25]. It should be noted that this model of injury, termed MLI, leads to posttraumatic OA that is detectable by 4 weeks after injury and progresses over a 4-month period, similar to that seen in the DMM model of posttraumatic OA [26]. The contralateral limb was sham-operated and represented the experimental control. Pre- and postsurgery mice were provided analgesia (intraperitoneal (IP) injection of 0.5 mg/kg buprenorphine) every 12 hours for 72 hours. Before killing mice at monthly intervals, the mice were fasted overnight, weighed, and tail-vein blood samples were taken for blood glucose measurements by using One Touch glucose meters (Lifescan, Inc., Milpitas, CA). Hemoglobin A1c measurements were made on tail-vein blood samples by using a Bayer DCA 2000 HgbA1C analyzer (Deerfield, IL). The assay is based on monoclonal antibody detection of the glycated amino terminus of the β-chain of human hemoglobin. The University Committee on Animal Resources approved all protocols.

### Tissue harvest and histologic analysis of OA progression

After establishment of deep anesthesia by using IP injection of 60 mg/kg ketamine and 4 mg/kg xylazine, mice were killed via cardiac perfusion by using 10 ml of phosphate-buffered saline followed by 10% neutral buffered formalin. Isolated knee joints were fixed for 3 days in 10% neutral buffered formalin at 23°C. Samples were decalcified for 2 weeks in 10% wt/vol EDTA and embedded in paraffin. Three-micrometer-thick sagittal sections from the medial joint compartment of the right knee were cut on a microtome and mounted on positively charged slides, baked at 60°C for 30 minutes, deparaffinized in xylene, and rehydrated in decreasing concentrations of ethanol.

Mouse knee-joint sections were stained with both Safranin O-fast green and Alcian blue-hematoxylin to allow visualization of cartilage tissue. Semiquantitative histopathologic grading of Alcian blue-stained knee-joint cartilage was performed on sagittal sections by using a murine scoring system established by the OARSI histopathology initiative [27], in which 0 = normal cartilage, 0.5 = loss of proteoglycan stain without cartilage damage, 1 = mild superficial fibrillation, 2 = fibrillation and/or clefting extending below the superficial zone, 3 = mild (< 25%) loss of cartilage, 4 = moderate (25% to 50%) loss of cartilage, 5 = severe (50% to 75%) loss of noncalcified cartilage, and 6 = eburnation with > 75% loss of cartilage.

### Quantification of cartilage morphologic features

After histologic grading, Alcian blue-stained sagittal sections were evaluated for cartilage area and thickness by using OsteoMeasure software (OsteoMetrics, Decatur, GA), as previously described [28]. Articular cartilage was defined as the area from the joint surface to the junction of the deep zone of the cartilage with the subchondral bone, including the entire surface from the anterior to the posterior edges of both the tibia and the femur. Cartilage area was quantified in each section by using an area-calculating algorithm in the software, as we previously described [25]. Similarly, tibial cartilage thickness was analyzed at the center of the tibial plateau. Osteophytes were positively identified by using the following criteria: the osteophyte (a) protruded into the joint space, (b) contained hypertrophic chondrocytes, and (c) displayed evidence of calcification. Histologic assessment of osteophytes was performed on each slide by using a digital projection at ×100 magnification. The largest cross-sectional dimension of the osteophyte was measured, as was the perpendicular dimension. These two determinations were averaged. Where multiple osteophytes were detected, the mean of the average osteophyte diameters was recorded. All three parameters (thickness, area, osteophyte size) were determined and averaged among all sections. Each diet group contained five to eight mice, with three sections from each joint being analyzed in the histologic grading and histomorphometric analyses.

### Micro-computed tomography (micro-CT) evaluation of mouse knee joints

Before histologic processing, knee joints were evaluated with micro-CT by using a Scanco vivaCT 40 scanner with 55-kVp source (Scanco USA, Inc., Wayne, PA). Joints were scanned at a resolution of 12 *μ*m, with a slice increment of 10 *μ*m. Images from each group were reconstructed at identical thresholds to allow 3-dimensional structural rendering of each joint. Analysis of bone volume was performed on selected regions between the femoral and tibial growth plates.

### Statistical analysis

Results are expressed as mean values ± SEM. Statistical analysis was performed by using StatView 5 software (SAS Institute, Cary, NC). Data were tested for normality by using the Kolmogorov-Smirnov test (with Dallal-Wilkinson-Lilliefor *P *value). Experimental means were compared by using ANOVA; sample means from three or more groups were compared, and the Fisher protected least significant differences test was used for comparisons between pairs of groups. A calculated *P *value of less than 0.05 was considered significant when comparing between-group differences.

## Results

**Figure 1:**
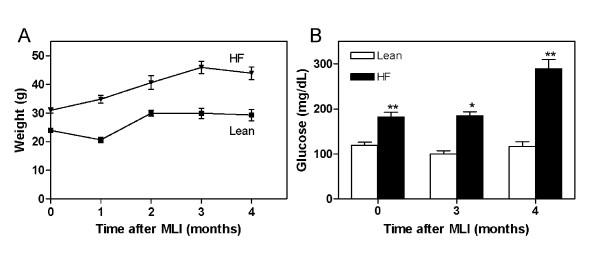
**High-fat diet promotes weight gain and hyperglycemia**. Five-week-old male C57BL/6 mice were placed on either a high-fat (60% kcal) or low-fat (10% kcal) diet for 2 months before meniscal-ligamentous injury (MLI). Body weight **(a) **and blood glucose levels **(b) **were determined at monthly intervals after MLI. *P < 0.05; **P < 0.01; *n *= 5 to 9 mice at each time point.

Five-week-old C57BL/6 mice were placed on either an HF diet representing 60% of total dietary kcal or an LF (lean) diet with 10% of kilocalories derived from fat. The HF diet has been shown to cause hyperglycemia and insulin resistance in this strain of mice [29]. After 8 weeks, mice on the HF diet weighed 29% more than those on the lean diet (30.9 ± 0.9 g versus 23.9 ± 0.6 g; *P *< 0.0001; Figure 1a). Fasting blood glucose levels were in the diabetic range only for the HF group (182.3 ± 10.5 mg/dl; Figure 1B), confirming a dysmetabolic state associated with the HF diet.

**Figure 2:**
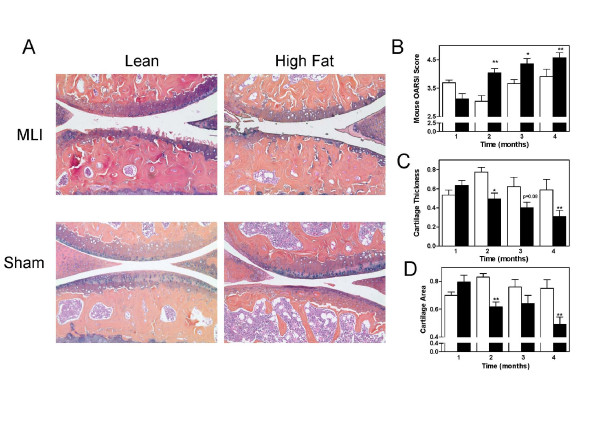
**Osteoarthritic changes were more pronounced in high-fat (HF) diet-treated mice**. **(a) **Histologic sections of injured and sham joints at 1-, 2-, 3-, and 4-month time points after meniscal-ligamentous injury (MLI) were stained with Alcian blue. Representative sections from the 4-month time point are shown. Magnification, ×100. **(b) **Slides were graded for OA severity by using the OARSI histopathology initiative scoring method for mice. The slides were also analyzed with histomorphometry for tibial cartilage thickness **(c) **and total articular cartilage area (tibia + femur, **d**). All histomorphometry data were normalized to sham-operated joint-cartilage thickness and area, respectively, from lean mice. All results are expressed as the mean ± SEM from at least five mice with three slides per mouse. *P < 0.05; **P < 0.01; *n *= 4 to 9 mice at each time point.

Progression to severe OA is typically observed within 5 months by using the meniscal-ligamentous injury (MLI) technique [25]. With the objective to determine whether the HF diet with its associated type 2 diabetic phenotype accelerates OA progression, mice from each dietary group (*n *= 4 to 9) were killed at monthly intervals out to 4 months after MLI surgery. Both groups gained weight over this 4-month period, with the HF group becoming markedly obese, with a peak weight of 45.9 ± 2.1 g compared with only 29.9 ± 1.8 g (*P *< 0.0001) for the lean group (Figure 1a). As expected, the HF group was diabetic (fasting blood glucose greater than 125 mg/dl), with peak levels of 288.8 ± 21.4 mg/dl at 4 months (Figure 1b). Histologic analysis of the articular cartilage at the 4-month time point revealed increased fibrillation, clefting, and decreased proteoglycan staining in response to MLI. These changes were more pronounced in the HF group (*P *< 0.01; Figure 2a) with major loss of cartilage. In some cases, this loss progressed nearly to the subchondral bone. No significant cartilage loss was observed in sham-operated knees of either HF or lean groups, with only occasional superficial zone fibrillation.

A progressive worsening of OA from 2 to 4 months was demonstrated in both diet groups, with the score for OA severity being significantly higher for the HF group at each of these time points (Figure 2b). Data for lean mice at month 1 indicate an unexpectedly advanced stage of OA for that time point. The explanation for this aberrant data point is not clear. With the OARSI score, the HF group on average had 25% to 50% loss of articular cartilage at 2 months (score of 4), whereas the lean group had less than 25% loss of articular cartilage at this same time point (score of 3; *P *< 0.01). As expected, the sham controls had scores of 1 or less, representing only occasional superficial zone fibrillation, even at 4 months (data not shown).

Histomorphometric analysis of the articular cartilage in the lean and HF groups over the 4-month time course confirmed the accelerated loss of articular cartilage in the HF diet group. Articular cartilage thickness on the tibial plateau progressively decreased in both groups but was approximately 40% to 50% thinner in the HF group between 2 and 4 months after MLI (Figure 2c). Similarly, total articular cartilage area, representing the combined cartilage areas of the femur and tibia, decreased progressively with the most marked difference between lean and HF diets being observed at the 4-month time point (Figure 2d). At this time point, cartilage area in the HF group had decreased by an additional 35% relative to lean controls (*P *< 0.01).

**Figure 3:**
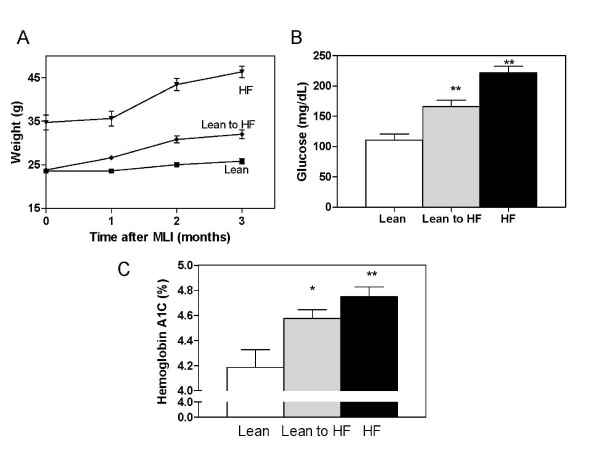
**Differing weight gain but similar hyperglycemia in short- and long-term high-fat (HF) diets**. Five-week-old male C57BL/6 mice were given either a high-fat (60% kcal) or low-fat (10% kcal) diet for 3 months before meniscal-ligamentous injury (MLI). After MLI, half of the lean group was switched to the HF diet. The three groups were maintained on diets for an additional 3 months. **(a) **Body weight was measured monthly after the MLI procedure. **(b) **Blood glucose and **(c) **hemoglobin A1c levels were determined at the 3-month end point. Data points represent mean ± SEM of at least six mice. *P < 0.05; **P < 0.01. *n *= 6 to 8 mice in each group.

In the preceding experiments, the HF-fed mice at the later time points were markedly obese in consequence of a 6-month diet protocol that included 2 months of the HF diet before the initiation of OA with MLI. To determine whether this marked increase in body weight was primarily responsible for the accelerated progression of OA with HF feeding, two HF diet protocols were compared. One group of mice (*n *= 6) was fed an HF diet for 3 months before MLI to establish obesity before initiation of OA. In a second group (*n *= 8), an HF diet was begun only at the initiation of OA. As a result of beginning the HF diet at the time of MLI, this group of mice demonstrated only a modest increase in body weight relative to the lean diet controls (*n *= 7) (Figure 3a). This contrasts with the long-term HF diet group, which again attained a marked obese body weight that was 80% above that of lean mouse controls. Despite the modest weight gain, the short-term HF group displayed metabolic dysfunction, as indicated by fasting hyperglycemia at 1-, 2-, and 3-month time points, although levels were less than those of the long-term HF group. Values at 3 months are shown (Figure 3b). An additional marker for a dysmetabolic state, hemoglobin A1c, a measure of average blood glucose levels over the preceding 3-month period [30], was nearly equally elevated in the two HF groups (Figure 3c). Note that the absolute hemoglobin A1c values were less than those of humans, for whom this immunoassay-based test is designed. These results indicate that the short-term HF diet produced metabolic dysfunction without requiring marked obesity or the extended 3-month period on the HF diet before the MLI surgery.

**Figure 4:**
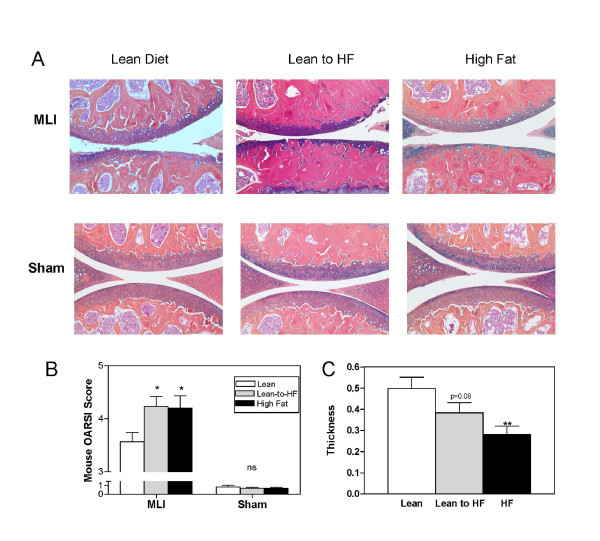
**OA progression after meniscal-ligamentous injury MLI is comparable in mice on either short- or long-term HF diets**. Injured and sham joints at 3 months after MLI were harvested from the lean, short-term HF, and long-term HF groups. **(a) **Histologic slides were stained with Alcian blue and **(b) **scored for OA severity by using the OARSI histopathology initiative scoring method for mice. The slides were also analyzed with histomorphometry for tibial cartilage thickness, with the data normalized to the cartilage thickness determined in sham-operated joints from lean mice **(c)**. Results are expressed as the mean ± SEM from at least six mice with three slides per mouse. *P < 0.05; **P < 0.01. *n *= 6 to 8 mice in each group.

Histologic analysis of the progression of OA at 3 months after MLI confirmed that a long-term HF diet was again associated with accelerated OA progression, with major loss of cartilage relative to the lean controls. In some cases, this loss progressed nearly to the subchondral bone. Importantly, the short-term HF diet group had an equally accelerated progression of OA when compared with the long-term HF group (Figure 4a). No significant cartilage loss was observed in sham-operated knees of either HF or lean groups, with only occasional superficial zone fibrillation. These observations were confirmed by comparably higher osteoarthritis grades for the HF groups relative to the lean controls, as assessed by the OARSI scoring method (*P *< 0.05; Figure 4b). It should be noted that the accelerated cartilage degeneration occurred in the short-term HF group despite the absence of marked obesity. In agreement with the OARSI scoring and supporting the idea that marked obesity is not required for acceleration of cartilage degeneration in mice fed an HF diet, tibial plateau articular cartilage thicknesses were 25% to 50% lower in the two HF groups compared with the lean group (Figure 4c). Cartilage-area determination supported the thickness data by demonstrating similar trends toward decreased area in both HF diet groups relative to mice fed the lean diet (data not shown). These results indicate that accelerated progression of OA in mice on an HF diet is associated with a diabetic metabolic phenotype, does not correlate with degree of weight gain, and does not require prolonged exposure to the HF diet.

**Figure 5:**
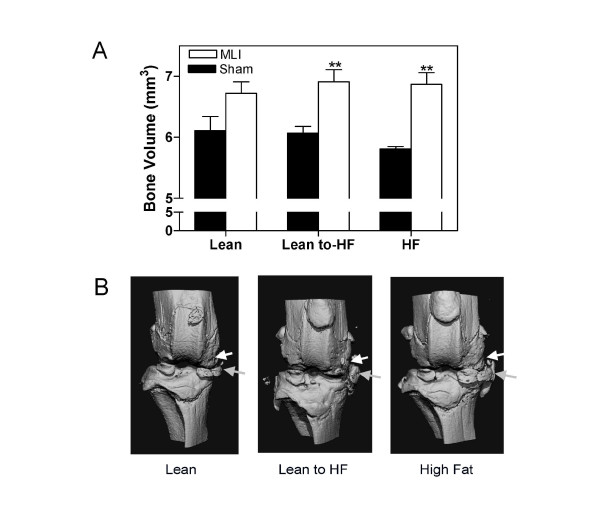
**Micro-CT analysis of knee joints after meniscal-ligamentous injury (MLI) as a function of diet**. **(a) **Knee-bone volumes of MLI and sham-treated limbs at 3 months in HF, Lean to HF, and Lean diet groups were analyzed with micro-CT. **(b) **Three-dimensional reconstructions of knee joints were used to visualize calcification of meniscus and osteophyte formation. Gray arrows, ossification of meniscus; white arrows, osteophyte-like structures. Representative 3D images are presented. Bone volume data represent the mean ± SEM of at least five mice. **P < 0.01. *n *= 6 to 8 mice in each group.

Quantitative micro-CT analysis of knee joints at the 3-month time point showed increased bone volume in the MLI knees relative to sham-treated controls, consistent with OA-like subchondral sclerosis and joint/periarticular mineralization. Only differences in the HF-diet groups, however, were statistically significant (*P *< 0.01; Figure 5a).

**Figure 6:**
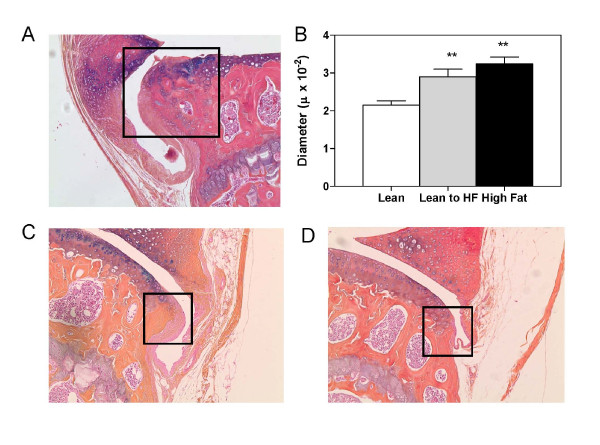
**Osteophyte analysis of knee joints after meniscal-ligamentous injury (MLI) as a function of diet**. **(a) **A representative osteophyte in a histologic section from an MLI-treated knee of an HF mouse is shown (boxed area). **(b) **Average osteophyte diameters on the tibial surface were measured by using digitally projected images of the histologic slides. Results are expressed as the mean ± SEM from at least six mice, with three slides per mouse. A representative osteophyte (boxed area) in a histologic section from a sham-operated knee of an HF mouse **(c) **and a comparable location in an unaffected lean diet control **(d) **are shown. ×100 magnification. **P < 0.01. *n *= 6 to 8 mice in each group.

Regarding enhanced joint and periarticular mineralization specifically, 3-dimensional reconstructions of the raw micro-CT data revealed a more progressive meniscal calcification and a suggestion of increased presence of osteophytes in both high-HF groups relative to the lean diet controls (Figure 5b). To investigate the possibility that HF diets promote the formation of osteophytes, all histologic slides were evaluated for the presence and size of osteophytes on the tibial surface. The blinded analysis revealed osteophytes in essentially all MLI-injured knees from all diet groups (Figure 6a). Importantly, not only was the size of the osteophytes greater in both HF-diet groups (*P *< 0.01), but they also were of comparable size in the short-term and long-term HF groups (Figure 6b). Interestingly, 30% of sham knees from HF-diet mice contained small osteophytes (Figure 6c), and another 20% showed early changes suggestive of osteophytes. This was distinct from sham knees from the lean mice, which did not display osteophytes, with early changes evident only in less than 20% of knees (Figure 6d). As with progression of cartilage degeneration and loss, more-aggressive osteophyte formation in MLI-injured knees from HF-diet mice is associated with a diabetic metabolic phenotype but does not correlate with degree of weight gain.

## Discussion

The results of this study demonstrate that consumption of a high-fat diet that leads to weight gain and diabetes will accelerate the progression of OA that is initiated by induction of a meniscal ligamentous injury. This effect on OA does not correlate, however, with degree of increase in body mass or exposure to a high-fat diet before injury. Our study draws different conclusions from the early work of Silberberg and Silberberg [31,32] who established a link between high-fat diets and OA in rodent models more than 50 years ago. In their pioneering work, they documented an increased incidence of OA in aged mice on an HF diet with age end points of 12 to 24 months [33]. Interestingly, a conclusion drawn from their data was that the incidence but not the rate of progression of OA was affected by the HF diet [9]. In our current study, we clearly establish that HF diets can accelerate the progression of OA after a defined OA initiation in a diarthrodial joint. Perhaps the variable time of spontaneous initiation, the long timeline of OA progression in the absence of a severe initiating injury, and the absence of quantitative end points may have contributed to the inability of Silberberg and Silberberg to detect changes in rate of progression in their studies. Alternatively, the effects of the HF diet on mechanisms of OA initiation and progression in aging versus acute injury may be different.

The relative contributions to OA of biomechanical factors resulting from increased body mass versus metabolic dysfunction in the obese and type 2 diabetic population have not been resolved. In humans, a strong correlation has been shown between elevated body mass index (BMI) and OA [21-23]. Biomechanical factors related to this increased body mass have been argued to make critical contributions to OA of the knee [34,35]. As for metabolic factors, correlations exist between diabetic parameters (hyperglycemia, hyperinsulinemia) and OA [36-39]. Another strong argument for systemic metabolic factors contributing to OA in the obese and diabetic population comes from the increased incidence of OA in the hand, a site not susceptible to the biomechanical overload present in the weight-bearing joints of an obese individual [37,38]. Logically, detrimental effects of body mass on OA would be a function of both magnitude of body-mass increase and length of exposure to this condition after initiation of OA. Our short-term HF diet group weighed the same as lean controls at the time of injury and gained approximately 6 g over the following 3-month period. In contrast, the long-term HF-diet group weighed more than 11 g more than controls at the time of knee injury and increased the differential to more than 20 g by 3 months. Despite this obvious difference in duration and degree of exposure to increased body mass, the two diet groups had comparable acceleration of OA progression. From our results, we conclude that weight gain does not correlate with progression of OA in our HF-diet mouse model. Silberberg and Silberberg [40] came to a similar conclusion that increased body weight in response to HF diets does not correlate with the incidence of joint disease in mice. Recently, Griffin *et al. *[41] reported the effect of HF diet-induced obesity on spontaneous OA of aging in female mice at 54 weeks of age and concluded that weight gain and loss of Safranin-O cartilage staining or OA severity score, but not cartilage degeneration, were correlated. Their histologic data suggested a modest OA progression with little loss of cartilage. Perhaps the early stages of OA initiation and progression are more strongly influenced by body mass. As cartilage degeneration becomes more severe, as in our MLI model, a high-fat diet continues to accelerate progression, and in this phase of the disease, the associated metabolic dysfunction becomes more important.

Silberberg and co-workers [42] did not investigate the metabolic dysfunction in their mouse models as a result of HF diets, but they did observe that the C57BL/6 strain but not the DBA strain was susceptible to increased OA of aging when on HF diets, despite weight gain in both strains. Sokoloff *et al. *[43,44] also observed that OA in the DBA strain was not altered by diet, despite weight gain. Recently, Griffin *et al. *[45] reported that markedly obese leptin-deficient mice do not have an increased incidence of OA. These observations provide further evidence that factors other than increased body mass are critical to the changes in OA in C57BL/6 mice. Interestingly, C57BL/6 mice are well known for their susceptibility to diabetic changes (hyperinsulinemia, hyperglycemia) when placed on HF diets, whereas DBA mice are resistant to these metabolic changes [29,46]. Although no cause and effect has been established, these observations show an association between development of a diabetic phenotype and accelerated progression of OA in the C57BL/6 mouse model.

In the mouse, metabolic changes in response to HF diets appear to be important in mediating osteoarthritic pathogenesis. Saturated fat diets derived from animal fat (lard) are most effective in mediating OA changes [31,44]. They are also more effective than dietary sugar in generating obesity and diabetes in experimental mice [47]. N-3 polyunsaturated fat (PUFA), such as linolenic acid, decreases the detrimental effects of the lard diet [48] and decrease expression and activity of proteoglycan-degrading enzymes and expression of inflammatory cytokines in articular chondrocyte cultures [49,50]. As a potential mechanism of action, the n-3 or omega-3 PUFAs that are found in fish oils have been shown to have anti-inflammatory effects with multiple mechanisms of action being proposed [51]. Importantly, one PUFA, eicosapentaenoic acid, has recently been shown to prevent and reverse insulin resistance in mice fed an HF diet [52]. The mechanism of this latter effect has been proposed to be suppression of adipose tissue inflammation [52]. These results suggest that HF diets mediate their effects on both OA and the diabetic phenotype, at least in part through inflammatory effects.

In relation to OA, the observation that obesity is a proinflammatory state may have critical implications for explaining both the increased progression of OA in our mouse model of obesity/type 2 diabetes and the epidemiologic data linking obesity to OA. The proinflammatory state of obesity may synergize with other OA-promoting pathways to accelerate the progression of the cartilage destruction. Thus, our laboratory is currently investigating the contribution of systemic inflammation to OA progression in obesity as a step toward elucidating novel candidate mechanisms that underlie the deleterious effect of the HF diet on the degenerative process.

## Conclusions

The results of this study indicate that an HF diet accelerates progression of OA in the C57BL/6 mouse diabetic model after a meniscal ligamentous injury, as assessed by both cartilage degeneration and osteophyte production. Degree of weight gain does not correlate with these changes, and prior exposure to the HF diet is not necessary for these effects. This suggests that metabolic dysregulation in type 2 diabetes is a comorbid factor in the progression of osteoarthritis, and its effect is in addition to that of obesity-related increased weight bearing in afflicted joints.

## Abbreviations

HF: high fat; HgbA1c: hemoglobin A1c; JNK: c-Jun N-terminal kinase; kcal: kilocalories; micro-CT: microcomputerized tomography; MLI: meniscal-ligamentous injury; OA: osteoarthritis; OARSI: Osteoarthritis Research Society International; PUFA: polyunsaturated fatty acid.

## Competing interests

The authors declare that they have no competing interests.

## Authors' contributions

RAM and MJZ conceived the study. RAM participated in the design and coordination of the experiments, carried out the animal studies, and drafted the manuscript. ERS performed the animal surgeries, participated in the scoring of histology slides and the coordination of the study, and helped draft the manuscript. JL performed the tissue processing, histology, histomorphometry, and data analysis. RNR participated in the design and data analysis. MJZ participated in the design and coordination of the experiments, participated in histologic scoring, and helped draft the manuscript. All authors read and approved the final manuscript.
